# *ACSL4* Expression Is Associated With CD8+ T Cell Infiltration and Immune Response in Bladder Cancer

**DOI:** 10.3389/fonc.2021.754845

**Published:** 2021-11-19

**Authors:** Wenjie Luo, Jin Wang, Xiaoyan Dai, Hailiang Zhang, Yuanyuan Qu, Wenjun Xiao, Dingwei Ye, Yiping Zhu

**Affiliations:** ^1^ Department of Urology, Fudan University Shanghai Cancer Center, Shanghai, China; ^2^ Department of Oncology, Shanghai Medical College, Fudan University, Shanghai, China; ^3^ Department of Urology, The First Affiliated Hospital of Shandong First Medical University, Shandong, China

**Keywords:** bladder cancer, immune checkpoint, *ACSL4*, CD8+ T cell, immunotherapy

## Abstract

**Objective:**

This study aimed to explore the role of *ACSL4* in CD8+ T cell tumor infiltration and outcomes of bladder cancer (BLCA) patients after immunotherapy.

**Methods:**

The correlation between *ACSL4* expression and tumor infiltration of immune cells was analyzed using the Tumor Immune Estimation Resource database. The prognostic significance of *ACSL4* in BLCA was analyzed using Kaplan–Meier curves. Immunohistochemistry was used to detect CD8+ T cell infiltration in tumors with high and low *ACSL4* expression obtained from patients at the Fudan University Shanghai Cancer Center. The relationships between immune checkpoint genes and immune response were analyzed using The Cancer Genome Atlas and IMvigor 210 cohorts. The molecular functions, cellular components, and biological processes involving *ACSL4* were explored using Kyoto Encyclopedia of Genes and Genomes and Gene Ontology enrichment pathway analyses.

**Results:**

The expression level of *ACSL4* was significantly correlated with the infiltration of CD8+ T cells in BLCA tumors (r = 0.192, P = 2.22e-04). Elevated *ACSL4* was associated with suppressed tumor progression and better outcomes for BLCA patients. The higher expression level of *ACSL4* predicted better immunotherapeutic responses and was associated with higher expression levels of core immune checkpoint genes, including *CD274*, *CTLA4*, *PDCD1*, and *LAG3*, compared with the low *ACSL4* expression group.

**Conclusion:**

This study demonstrated for the first time that elevated *ACSL4* correlated significantly with CD8+ T cell infiltration and contributed to better immunotherapeutic responses in BLCA patients. Furthermore, *ACSL4* serves as a novel biomarker for predicting patient outcomes after immunotherapeutic treatments, which may improve the development of individualized immunotherapy for BLCA.

## Introduction

Bladder cancer (BLCA) is the ninth most common malignant tumor worldwide, with approximately 81,400 new cases and 17,980 deaths reported in the United States in the year 2020 (1). Routine treatments for BLCA, such as platinum-based chemotherapy and intravesical bacillus Calmette-Guerin, frequently fail because of the biological behavior of malignant progression and high recurrence rate after treatment (2). According to the latest reports, the median overall survival of patients with relapsed or refractory BLCA after cisplatin treatment was only 14–15 months (3). In recent years, immune checkpoint inhibitor (ICI) therapies, especially anti-programmed cell death protein 1 (PD-1), anti-PD-ligand 1 (PD-L1), and anti-cytotoxic T lymphocyte-associated antigen 4 (CTLA4) antibodies, have achieved significant success in BLCA treatment. The United States Food and Drug Administration has approved five PD-1/PD-L1 inhibitors as first- or second-line treatments for patients with advanced BLCA (4). However, among patients with advanced BLCA, the overall response rates for ICI treatments are 13–24% (5, 6). Because the majority of advanced BLCA patients do not benefit from these immunotherapeutic agents, it is important to identify new biomarkers for predicting treatment response.

Acyl-CoA synthetase long-chain family member 4 (*ACSL4*) has been recognized as an important molecule in metabolism-associated diseases (7). Furthermore, *ACSL4* was reported to promote the esterification of arachidonoyl and adrenoyl into phosphatidylethanolamine, which is a process closely related to ferroptosis (8, 9). Intriguingly, recent evidence showed that ferroptosis-inducing therapy was potentiated by anti-PD-L1 antibodies. Specifically, anti-PD-L1 antibodies stimulated CD8+ T cells to secrete interferon γ, which suppressed the glutamate–cystine antiporter system in target cancer cells and sensitized them to ferroptosis (10). Therefore, immunotherapy in combination with ferroptosis induction represents a promising treatment because the two therapeutic modalities may mutually potentiate each other and lead to a synergistic anticancer effect.

As the main effector immune cells, CD8+ T cells play a critical role in preventing tumor occurrence and development (11). It has been reported that the populations of intratumoral CD8+ T cells are highly heterogeneous (12). Preclinical models have indicated that infiltration of CD8+ T-cells in tumors is strongly associated with anti-PD-1/PD-L1 treatment (12, 13). In the current study, we demonstrated that the expression level of *ACSL4* was positively correlated with the infiltration of CD8+ T-cells in BLCA. Furthermore, *ACSL4* was associated with the expression of immune checkpoint genes and may represent a predictive biomarker for anti-PD-1/PD-L1 treatment. This study is the first exploration of the comprehensive clinical value and immunological implication of *ACSL4* in BLCA.

## Materials and Methods

### Data Collection

We downloaded the BLCA gene expression profile of the Cancer Genome Atlas (TCGA) database from UCSC Xena (https://tcga.xenahubs.net, version of data: 2019-12-06). For validation, we enrolled a total of 30 BLCA patients at the Fudan University Shanghai Cancer Center (FUSCC, Shanghai, China) from August 2019 to May 2021. Our study was approved by the Ethics Committee of FUSCC. Informed consent was obtained from all patients who participated in this study. To discover the role of *ACSL4* in BLCA immunotherapy, we obtained the genetic profiles of 195 BLCA patients from the IMvigor 210 cohort (http://research-pub.gene.com/IMvigor210CoreBiologies/, accession number: EGAS00001002556), who underwent treatment with the PD-L1 inhibitor atezolizumab with documented ICI responsiveness (14).

### Kaplan–Meier Survival Curve Analysis

Based on the analysis of hazard ratios (HR) and log-rank P-values, Kaplan–Meier plots (http://kmplot.com/analysis/) were used to analyze the relationship between *ACSL4* gene expression and survival rates in the TCGA BLCA cohort in combination with restricted analysis of cellular content (enriched or depleted CD8+ T cells) (15).

### TIMER Analysis

To analyze the tumor-infiltrating immune cells in pan-cancers, we used the Tumor Immune Estimation Resource (TIMER) database (https://cistrome.shinyapps.io/timer/, version: 2.0) and >10,000 samples from the TCGA database (16). TIMER analysis was performed to obtain the abundance of tumor-infiltrating immune cells based on the statistical analysis of gene expression profiles (17). The correlations between the expression level of *ACSL4* and infiltrating immune cells, including CD4+, CD8+, regulatory T cells, B cells, neutrophils, dendritic cells, and M1 and M2 macrophages, were analyzed based on the expression of specific immune cell-related marker genes in BLCA. The marker genes of tumor-infiltrating immune cells were based on data from previous studies (18, 19). The associations between mRNA expression levels of *CD274* (*PD-L1*), *CTLA4*, *LAG3*, and *PDCD1* (*PD-1*) and the expression levels of *ACSL4* and CD8 cell markers, including *CD8A* and *CD8B* from the TCGA BLCA cohort, were determined using the TIMER database. The expression levels of *ACSL4*, *CD8A*, and *CD8B* genes were represented on the x-axes, and related marker genes were placed on the y-axes.

### Immunohistochemistry Staining and Evaluation

IHC was performed on formalin-fixed, paraffin-embedded tissues obtained from patients with BLCA. The primary antibodies used for the detection of the targeted proteins were anti-*ACSL4* (Clone OTI6B7, NOVUS, Dilution: 1:500) and anti-CD8 (Clone 66868, Proteintech, Dilution: 1:2,000). The positive cells were enumerated from the representative views in high-power field [high-power field (HPF), 40×, objective], and the mean value was adopted. For quantification of protein, positive and negative images of the IHC specimens were acquired and analyzed using the IHC Profiler in Image J software (NIH, Bethesda, MD, USA). All samples were evaluated by two independent, experienced pathologists.

### Functional Analysis of *ACSL4*

Protein–protein interactions for *ACSL4* were predicted using the STRING database (https://string-db.org, version:11.5) (20). Kyoto Encyclopedia of Genes and Genomes (KEGG) and Gene Ontology enrichment pathway analyses were performed to evaluate molecular functions, cellular components, and biological processes involving *ACSL4*. To illustrate biological functions of prognostic genes in high-risk and low-risk patient groups, Gene Set Enrichment Analysis was explored to identify pathways and was based on TCGA data (21).

### Statistics

The figures were partially drawn by GraphPad Prism 8.0 software (GraphPad Software Inc.). Two-tailed Student’s t-test or One-way ANOVA was used to measure differences between groups. P < 0.05 was considered statistically significant.

## Results

### The Level of *ACSL4* Expression Is Positively Correlated With the Infiltration Level of CD8+ T Cell in BLCA

**Figure 1:**
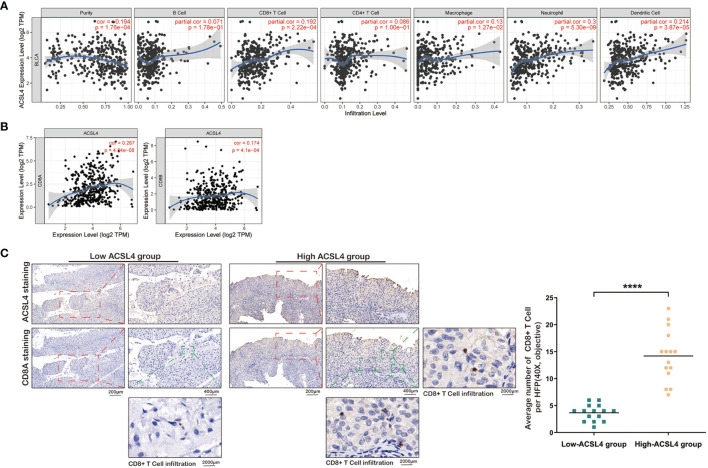
Elevated *ACSL4* is associated with CD8+ T Cell infiltration. **(A)** The correlations between six kinds of immune cell, immune purity, and expression level of *ACSL4* were identified from TIMER analysis. **(B)** The expression level of *ACSL4* was proportional to the expression level of *ACSL4*. **(C)** IHC of *ACSL4* and CD8A staining detected the CD8+ T Cell infiltration in low-*ACSL4* group and high-*ACSL4* group. The red box stands for a representative image of *ACSL4* or CD8A staining. The green box stands for the observed CD8+ T Cells. Results are presented as mean ± SD. ****P < 0.0001. Data were obtained from three independent experiments.

As shown in **Figure 1A**, the level of *ACSL4* expression positively correlated with immune purity (R = 0.194, P = 1.76e-04) and the infiltration levels of specific subsets of immune cells, including B cells (R = 0.071, P = 1.78e-01), CD8+ T cells (R = 0.192, P = 2.22e-04), CD4+ T cells (R = 0.086, P = 1.00e-01), macrophages (R = 0.13, P = 1.27e-02), neutrophils (R = 0.3, P = 5.30e-09), and dendritic cells (R = 0.2140, P = 3.87e-05) in BLCA. The correlation between *ACSL4* expression level and immune cells in pan-cancers is presented in **Supplementary Figure 1**. The expression level of *ACSL4* was positively correlated with the expression of *CD8A* (R = 0.174, P = 4.1e-04) and *CD8B* (R = 0.267, P = 4.84e-08) marker genes, further confirming a role for *ACSL4* in CD8+ T cell infiltration (**Figure 1B**). And the correlation analysis between *ACSL4* and other immune cell–related markers is presented in **Table 1**. The 30 patients from our Cancer Center were divided into high- and low-*ACSL4*-expression groups (15 samples each) for IHC analysis. As shown in **Figure 1C**, the average number of CD8+ T cells (HFP, 40×, objective) in the high-*ACSL4*-expression group was significantly greater than that in the low-*ACSL4*-expression group.

**Table 1:** Correlation analysis between *ACSL4* and immune cell–related markers in TCGA BLCA cohort.

Description	Markers	None		Tumor purity		Age	
		Correlation	P-value	Correlation	P-value	Correlation	P-value
CD8+ T cell	CD8A	0.267	**4.84E-08**	0.188	**2.94E-04**	0.268	**4.19E-08**
	CD8B	0.174	**4.10E-04**	0.108	**3.83E-02**	0.174	**4.17E-04**
T cell (general)	CD3E	0.264	**9.50E-09**	0.165	**1.53E-03**	0.265	**6.15E-08**
	CD3D	0.201	**4.41E-05**	0.092	7.68E-02	0.202	**4.09E-05**
	CD2	0.257	**1.64E-07**	0.159	**2.26E-03**	0.258	**1.40E-07**
B cell	CD19	0.086	8.12E-02	−0.054	3.01E-01	0.085	8.89E-02
	CD79A	0.149	**2.57E-03**	0.017	7.44E-01	0.148	**2.85E-03**
M1 Macrophage	iNOS	−0.193	**9.01E-05**	−0.161	**1.96E-02**	−0.193	**8.73E-05**
	IRF5	−0.032	5.13E-01	−0.055	2.88E-01	−0.033	5.13E-01
	COX2	0.249	**3.76E-07**	0.237	**4.44E-06**	0.251	**3.16E-07**
M2 Macrophage	CD163	0.326	**1.92E-11**	0.256	**6.64E-07**	0.326	**1.55E-11**
	VSIG4	0.323	**2.94E-11**	0.254	**8.11E-07**	0.323	**2.56E-11**
Neutrophils	CD66b	0.054	2.78E-01	0.05	3.36E-01	0.054	2.78E-01
	CD11b	0.295	**1.57E-09**	0.179	**5.41E-04**	0.294	**1.52E-09**
	CCR7	0.073	1.43E-01	0.014	7.85E-01	0.071	1.52E-01
	MS4A4A	0.318	**6.29E-11**	0.231	**7.52E-06**	0.318	**5.57E-11**
Dendritic cell	HLA-DPB1	0.287	**4.21E-09**	0.202	**9.56E-05**	0.286	**4.20E-09**
	HLA-DQB1	0.277	**1.26E-08**	0.276	**3.94E-04**	0.184	**1.48E-08**
	HLA-DRA	0.333	**7.02E-12**	0.272	**1.17E-07**	0.333	**5.95E-12**
	HLA-DPA1	0.313	**9.67E-11**	0.249	**1.38E-06**	0.313	**1.08E-10**
Treg	FOXP3	0.344	**1.29E-12**	0.283	**3.36E-08**	0.346	**7.80E-13**
	CCR8	0.415	**2.14E-18**	0.372	**1.60E-13**	0.416	**1.85E-18**
	TGFβ	0.166	**7.63E-04**	0.14	**7.13E-03**	0.167	**7.35E-04**

P-value < 0.05 is highlighted using bold font.

### Dysregulated Expression of *ACSL4* in Patients With Tumor *In Situ* and Non-Muscle-Invasive or Muscle-Invasive BLCA

**Figure 2:**
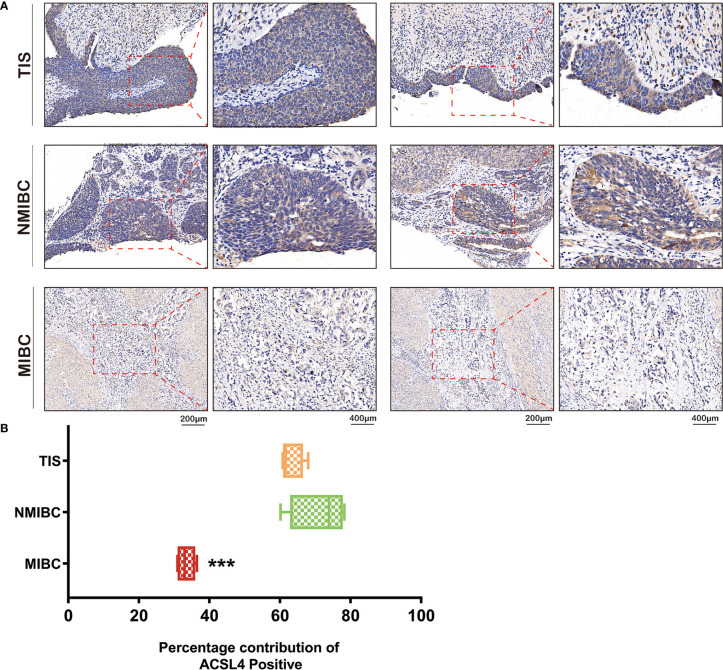
Different expression of *ACSL4* in TIS, NMIBC, and MIBC. **(A)** IHC of *ACSL4* staining evaluated the expression level of *ACSL4* in TIS, NMIBC, and MIBC. **(B)** Analysis of Image J IHC Profiler indicated expression level of *ACSL4* in MIBC was significantly lower compared with that in TIS or NMIBC. (P < 0.001). The red box stands for a representative image of *ACSL4* staining. Results are presented as mean ± SD. ***P < 0.001. Data were obtained from three independent experiments.

Immune cell infiltration is tightly associated with the invasive ability of tumors. Therefore, we applied IHC analysis to detect *ACSL4* expression in tumors from patients diagnosed with different types of BLCA (**Figure 2A**). The results showed that *ACSL4* expression was significantly higher in tumor *in situ* (TIS) and non-muscle-invasive BLCA (NMIBC) compared with muscle-invasive BLCA (MIBC), which suggested that *ACSL4* may play a role in preventing BLCA invasion by facilitating immune cell infiltration (**Figure 2B**).

### The Prognostic Significance of *ACSL4* Expression in BLCA Patients With Enriched or Depleted CD8+ T Cells

**Figure 3:**
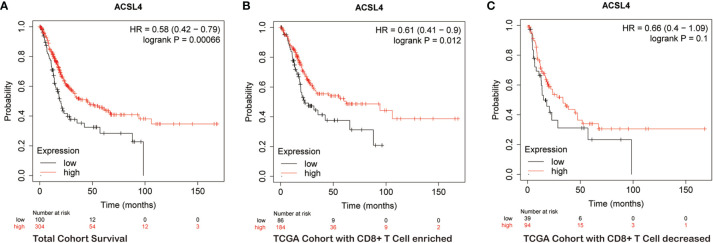
*ACSL4* is significantly associated with prognosis of BLCA patients. **(A–C)** Kaplan-Meier plots evaluated the overall survival of patients in total TCGA BLCA cohort and cohorts with CD8+ T Cell enriched or decreased.

Next, we explored the prognostic value of *ACSL4* for BLCA patients because there is a strong association between immune infiltration, tumor invasion, and patient survival. Kaplan–Meier analysis showed that patients with high *ACSL4* expression had a significantly better overall survival compared with patients with low *ACSL4* expression (P = 6.6e-04) (**Figure 3A**). Furthermore, we combined survival analysis with CD8+ T cell enrichment. In the CD8+ T cell-enriched cohort, the overall survival of patients with high *ACSL4* expression was greater compared with patients with low *ACSL4* expression (P = 0.012), while no significant differences were detected in the CD8− T cell-enriched cohort (P = 0.1) (**Figures 3B, C**). These results suggested that high expression of *ACSL4* may cooperate synergistically with infiltration of CD8+ T cells. The clinicopathological characteristics of enrolled cohorts are presented in **Supplementary Table 2**.

### The Role of *ACSL4* and CD8+ T Cell Infiltration in the Expression of Immune Checkpoint-Related Genes and Immunotherapy Response

**Figure 4:**
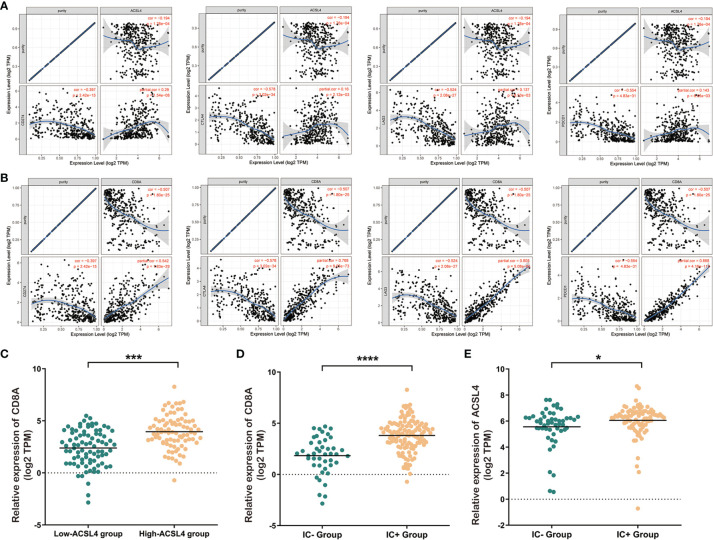
Elevated expression level of *ACSL4* is associated with better immunotherapeutic response. **(A)** TIMER analysis showed the positive expression correlations between *ACSL4* and immune checkpoint genes including CD274 (PD-L1), CTLA-4, LAG-3, and PDCD1 (PD-1). **(B)** TIMER analysis showed the positive expression correlations between CD8A and immune checkpoint genes including CD274 (PD-L1), CTLA-4, LAG-3, and PDCD1 (PD-1). **(C)** Dysregulated expression of CD8A in low-*ACSL4* group and high-*ACSL4* group from IMvigor 210 cohort. **(D, E)** The immune cell response-positive (IC+) group showed significantly higher expression level of CD8A (P < 0.0001) and *ACSL4* (P<0.05). Results are presented as mean ± SD. *P < 0.05; ***P < 0.001, ****P < 0.0001. Data were obtained from three independent experiments.

To further investigate the clinical significance of *ACSL4* and CD8+ T cell infiltration, we explored the associations between *ACSL4* and *CD8A* expression and expression of immune checkpoint genes, which are important markers for BLCA immunotherapy responses. As shown in **Figures 4A, B**, the expression of the immune checkpoint-related genes *CD274*, *CTLA4*, *LAG3*, and *PDCD1* positively correlated with *ACSL4* expression. Subsequently, to validate the role of *ACSL4* in response to immunotherapy, we applied the correlation analysis to the IMvigor 210 cohort. The results confirmed that the level of *CD8A* mRNA was significantly greater in the high-*ACSL4*-expression group compared with the low-*ACSL4*-expression group (**Figure 4C**). Both *ACSL4* and *CD8A* mRNA levels were significantly higher in the immunotherapy responsive group compared with the non-responsive group (**Figures 4D, E** **)**. Together, these findings indicated that the upregulation of *ACSL4* was associated with increased infiltration of CD8+ T cells and, subsequently, facilitated the expression of immune checkpoint-related genes, which improved response to immunotherapy in BLCA.

### Mechanistic Analysis of *ACSL4* in BLCA

**Figure 5:**
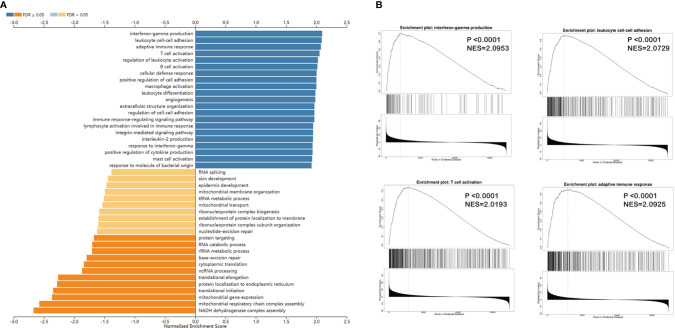
Immune checkpoints genes are expressed differently in BCa groups. **(A)** GSEA analysis showed the related biological processes of *ACSL4* in TCGA cohort. **(B)** The top four of related processes were interferon-gamma production (P <0.0001, NES=2.0953), adaptive immune response (P <0.0001, NES=2.0925), leukocyte cell-cell adhesion (P <0.0001, NES=2.0729), and T cell activation (P <0.0001, NES=2.0193).

**Figure 6:**
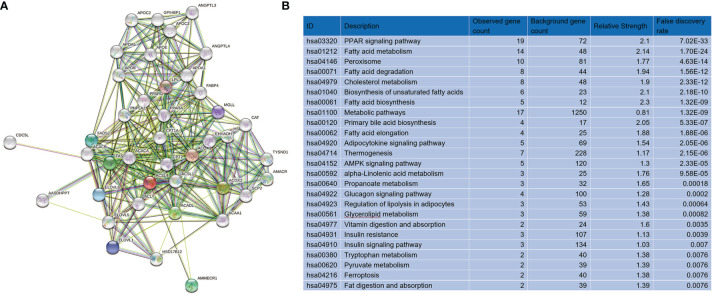
Targeted genes and molecular pathways of *ACSL4*. **(A)** PPI network presented the targeted genes of *ACSL4*. **(B)** KEGG analysis showed the top 25 molecular pathways correlated with *ACSL4*.

We applied Gene Set Enrichment Analysis to predict functional changes between the high- and low-*ACSL4*-expression groups (**Figure 5A**). This biological analysis for *ACSL4* indicated that the top four enriched pathways were interferon γ production (P < 0.0001, normalized enrichment score [NES] = 2.0953), adaptive immune response (P < 0.0001, NES = 2.0925), leukocyte cell-cell adhesion (P < 0.0001, NES = 2.0729), and T cell activation (P < 0.0001, NES = 2.0193), which were consistent with results from the TIMER analysis (**Figure 5B**). Furthermore, we performed protein-protein interaction analysis to acquire targeted proteins of *ACSL4*, which included ACSL1, ACACA, FASN, PPARG, and PPARA (**Figure 6A**). As shown in **Figure 6B**, KEGG analysis indicated that *ACSL4*-correlated genes were mainly located in metabolic pathways, including the PPAR signaling pathway (false discovery rate [FDR] = 7.02e-33), fatty acid metabolism (FDR = 1.70e-24), fatty acid degradation (FDR = 1.56e-12), and cholesterol metabolism (FDR = 2.33e-12). Gene Ontology analysis of *ACSL4* including Cellular Component, Molecular Function, and Biological Process is shown in **Supplementary Table 1**, which confirmed the tight association between *ACSL4* expression and immune cell infiltration.

## Discussion

The traditional treatments for bladder cancer have not significantly improved the survival rates of patients. Recent studies have shown that immunotherapies based on immune checkpoint blockage, such as anti-PD-1/PD-L1 and anti-CTLA-4 antibodies, have prominent efficacy against bladder cancer (22, 23). However, immunotherapy lacks sufficient biomarkers because the majority of BLCA patients develop a negative antitumor immune response. Recent studies have discovered more and more immune-related genes in regulating important phenotypes through controlling different pathways in multiple cancers (24, 25). For example, BRCA1-associated protein was shown to regulate liver hepatocellular patients’ prognosis *via* immune cell infiltration (26). In the current study, we found that the expression level of *ACSL4* in BLCA was positively correlated with tumor infiltration of CD8+ T cells, which may affect the efficacy of immunotherapy in BLCA patients.

Recently, there has been renewed interest in the regulation of *ACSL4* in oncology research because this protein plays a vital role as a hub gene in metabolism and ferroptosis of tumor cells (8, 27). For example, *ACSL4* was shown to facilitate hepatocellular carcinoma (HCC) development and modulate aberrant lipid metabolism *via* the c-MYC/SREBP1 pathway (28). Our study found a tight association between *ACSL4* and tumor-related lymphocytes, including CD8+ T cells. Our results reflect those of Liu et al. (29), who confirmed the correlation between metabolism and the immune response. Furthermore, these authors found that the metabolic regulator fat mass- and obesity-associated protein was utilized by tumors to escape immune surveillance, which suppressed the checkpoint blockade and immunotherapeutic responsiveness (29).

We discovered that *ACSL4*, which meditated CD8+ T cell infiltration, was associated with tumor invasiveness. This observation is consistent with that of Li et al., who found that tumor metastasis was facilitated by elevated miR-301a levels, the latter of which correlated with subsequent antitumor-immunity and suppression of CD8+ T cell recruitment (30). Several studies have reported that tumor invasion and immune environment play significant roles in survival outcome and immunotherapy response in cancer patients (31–33). We propose that determining the expression levels of *ACSL4* and status of CD8+ T cell infiltration may be useful for clinicians to better predict the prognosis of patients who undergo BLCA immunotherapy.

Multiple studies have confirmed that the infiltration and effector function of CD8+ T cells in the tumor micro-environment can be enhanced by effective cancer immuno-therapy (34–36). Philip and Schietinger recognized that predicting which patients will respond to immunotherapy is an important challenge and understanding CD8 T cell differentiation and dysfunction will be key to mediating a clinical response (37). From the analysis of the IMvigor 210 cohort in our study, we validated that the tight correlation between CD8+ T cell infiltration and expression level of *ACSL4* contributed to the immune response in BLCA patients who underwent immunotherapy.

We explored the mechanisms related to the immunological role of *ACSL4* in BLCA. The Gene Set Enrichment Analysis of tumors from BLCA patients confirmed that the immune cell recruitment and response mediated by *ACSL4* was consistent with the TIMER database analysis. Protein-protein interaction, KEGG, and Gene Ontology analyses indicated that metabolic regulation of tumors by *ACLS4* contributed to immunological responsiveness and the immunotherapeutic outcome of BLCA patients. This finding broadly supports the work of Vantaku et al., who linked tumor metabolism with progression of BLCA (38), and the review by Afonso et al. that focused on the role of metabolism in immunotherapeutic efficacy of ICIs used for treating BLCA patients. Afonso et al. found that molecular hallmarks of cancer cell metabolism suppressed malignant cells, facilitated immunotherapeutic responses, and represented potential therapeutic targets (39). Recently, the role of *ACSL4* in other types of tumors has been reported, especially in HCC (40–42). *ACSL4* modulated aberrant lipid metabolism (28) and survival outcome of HCC patients and was validated as a predictive biomarker of sorafenib-induced ferroptosis in HCC (43).

In conclusion, for the first time, we revealed a potential immunotherapeutic function for *ACSL4* in BLCA that may play a role in ICI interventions. This study demonstrated that *ACSL4* correlated significantly with the recruitment of immune cells, including critical CD8+ T cells, in the BLCA microenvironment, which may have prevented tumor invasion and improved survival outcomes for BLCA patients. However, more evidence and validation from multiple cohorts remains to be further investigated. The current study indicated that *ACSL4* as a biomarker may be useful for predicting outcomes of patients after immunotherapeutic treatments and may have important translational impacts in the development of precise therapy for BLCA.

## Data Availability Statement

The datasets presented in this study can be found in online repositories. The names of the repository/repositories and accession number(s) can be found below: https://www.ncbi.nlm.nih.gov/, 10.1038/nature25501.

## Ethics Statement

The studies involving human participants were reviewed and approved by Fudan University Shanghai Cancer Center Ethics Committee. The patients/participants provided their written informed consent to participate in this study.

## Author Contributions

The work presented here was carried out in collaboration among all authors. YZ, DY, and WL defined the theme of the study and discussed analysis, interpretation, and presentation. WL and YQ drafted the manuscript, analyzed the data, developed the algorithm, and explained the results. WX and WL participated in the collection of relevant data and helped draft the manuscript. JW and XD helped to perform the statistical analysis. DY and HZ helped revise the manuscript and provided guiding suggestions. All authors contributed to the article and approved the submitted version.

## Funding

This work is supported by grants from the National Natural Science Foundation of China (No. 81772706 and No. 81802525).

## Conflict of Interest

The authors declare that the research was conducted in the absence of any commercial or financial relationships that could be construed as a potential conflict of interest.

## Publisher’s Note

All claims expressed in this article are solely those of the authors and do not necessarily represent those of their affiliated organizations, or those of the publisher, the editors and the reviewers. Any product that may be evaluated in this article, or claim that may be made by its manufacturer, is not guaranteed or endorsed by the publisher.

## Supplementary Material

Supplementary Figure 1 Landscape of *ACSL4* in correlation with immune cell infiltration in pan-cancers

## Abbreviations

BLCA, bladder cancer; ICI, immune checkpoint inhibitor; PD-1, programmed cell death protein 1; PD-L1, programmed cell death protein-ligand 1; CTLA4, cytotoxic T lymphocyte-associated antigen 4; *ACSL4*, Acyl-CoA synthetase long-chain family member 4; TCGA, The Cancer Genome Atlas; HR, hazard ratios; TIMER, Tumor Immune Estimation Resource; KEGG, Kyoto Encyclopedia of Genes and Genomes; IHC, Immunohistochemistry; HPF, high-power field; TIS, tumor *in situ* bladder cancer; NMIBC, non-muscle-invasive bladder cancer; MIBC, muscle-invasive bladder cancer.
