# Supplementary figures and images for: ACSL4 Expression Is Associated With CD8+ T Cell Infiltration and Immune Response in Bladder Cancer
Source: Front Oncol. 2021 Nov 19;11:754845. doi: 10.3389/fonc.2021.754845 (PMC8640077; doi:10.3389/fonc.2021.754845)

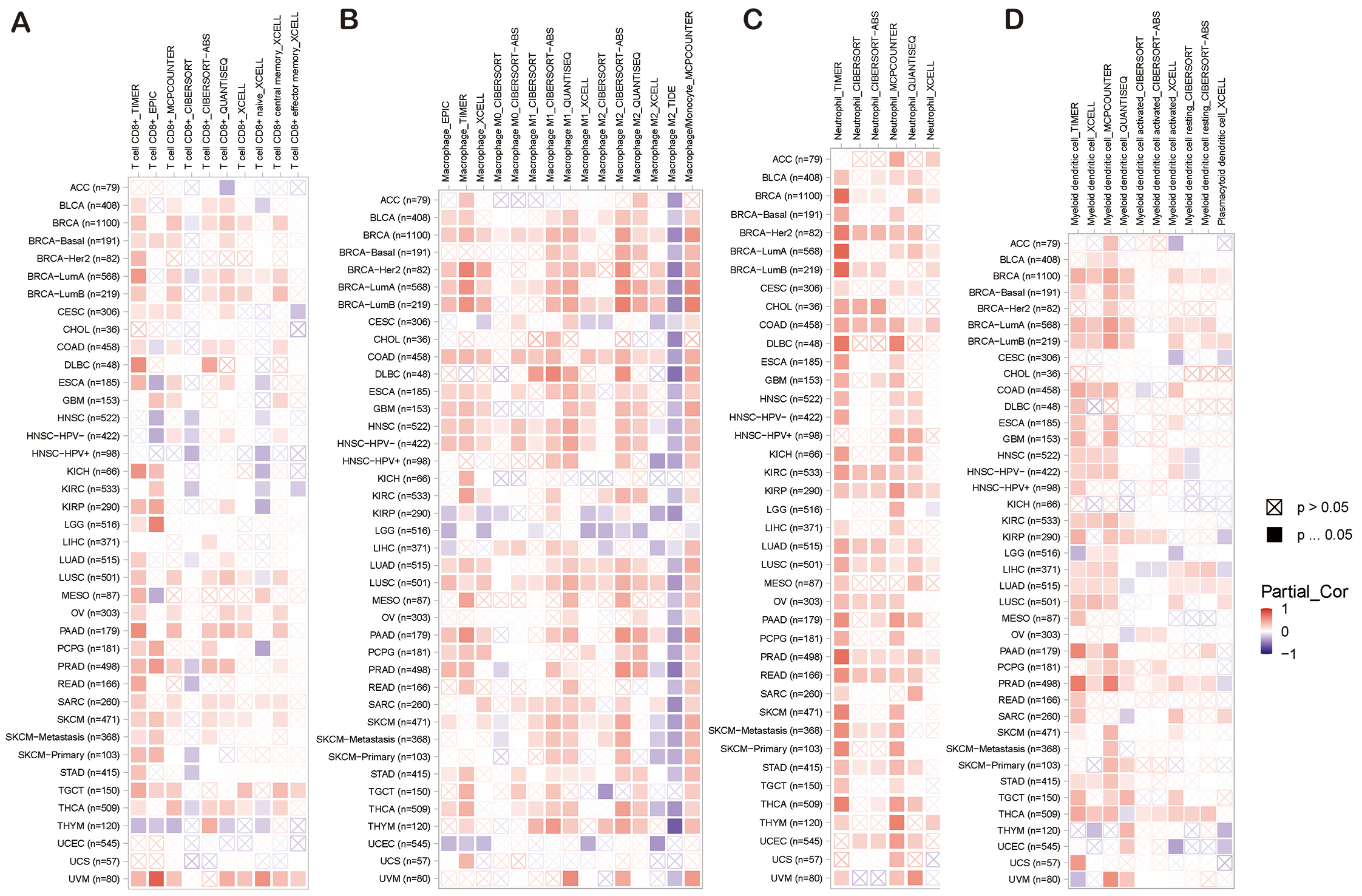

Supplement: Supplementary Figure 1 — Landscape of ACSL4 in correlation with immune cell infiltration in pan-cancers [file Image_1.jpeg]
