# Supplementary material for: ACSL4 Expression Is Associated With CD8+ T Cell Infiltration and Immune Response in Bladder Cancer
Source: Front Oncol. 2021 Nov 19;11:754845. doi: 10.3389/fonc.2021.754845 (PMC8640077; doi:10.3389/fonc.2021.754845)
Supplement: Supplementary file 2 [file Table_1.docx]

Table S1 Gene Ontology analysis of ACSL4 including Cellular Component, Molecular Function and Biological Process.

| Gene Ontology analysis of Cellular Component | | | | | |
| --- | --- | --- | --- | --- | --- |
| Term ID | Term description | Observed gene count | Background gene count | Strength | False discovery rate |
| GO:0005777 | peroxisome | 11 | 127 | 1.62 | 5.91E-13 |
| GO:0005782 | peroxisomal matrix | 9 | 53 | 1.91 | 5.91E-13 |
| GO:0042627 | chylomicron | 7 | 13 | 2.41 | 5.91E-13 |
| GO:0034358 | plasma lipoprotein particle | 8 | 40 | 1.98 | 1.67E-12 |
| GO:0034361 | very-low-density lipoprotein particle | 7 | 20 | 2.22 | 1.75E-12 |
| GO:0034363 | intermediate-density lipoprotein particle | 5 | 6 | 2.6 | 2.07E-10 |
| GO:0034364 | high-density lipoprotein particle | 6 | 28 | 2.01 | 9.80E-10 |
| GO:0034362 | low-density lipoprotein particle | 4 | 14 | 2.13 | 6.07E-07 |
| GO:0034366 | spherical high-density lipoprotein particle | 3 | 9 | 2.2 | 2.11E-05 |
| GO:0005778 | peroxisomal membrane | 4 | 51 | 1.57 | 5.24E-05 |
| Gene Ontology analysis of Molecular Function | | | | | |
| Term ID | Term description | Observed gene count | Background gene count | Strength | False discovery rate |
| GO:0016746 | transferase activity, transferring acyl groups | 10 | 212 | 1.35 | 7.21E-09 |
| GO:0016747 | transferase activity, transferring acyl groups other than amino-acyl groups | 9 | 179 | 1.38 | 2.35E-08 |
| GO:1901681 | sulfur compound binding | 9 | 234 | 1.26 | 1.52E-07 |
| GO:0003997 | acyl-CoA oxidase activity | 4 | 6 | 2.5 | 2.64E-07 |
| GO:0015248 | sterol transporter activity | 5 | 28 | 1.93 | 3.71E-07 |
| GO:0070325 | lipoprotein particle receptor binding | 5 | 27 | 1.95 | 3.71E-07 |
| GO:0008289 | lipid binding | 12 | 673 | 0.93 | 4.13E-07 |
| GO:0004312 | fatty acid synthase activity | 4 | 11 | 2.24 | 7.58E-07 |
| GO:0005102 | signaling receptor binding | 16 | 1513 | 0.7 | 8.45E-07 |
| GO:0015485 | cholesterol binding | 5 | 45 | 1.72 | 1.72E-06 |
| Gene Ontology analysis of Biological Process | | | | | |
| Term ID | Term description | Observed gene count | Background gene count | Strength | False discovery rate |
| GO:0044255 | cellular lipid metabolic process | 35 | 946 | 1.25 | 8.77E-37 |
| GO:0006631 | fatty acid metabolic process | 26 | 294 | 1.63 | 4.86E-34 |
| GO:0032787 | monocarboxylic acid metabolic process | 27 | 477 | 1.43 | 5.83E-31 |
| GO:0044242 | cellular lipid catabolic process | 20 | 167 | 1.76 | 9.70E-28 |
| GO:0019216 | regulation of lipid metabolic process | 23 | 373 | 1.47 | 2.10E-26 |
| GO:0016042 | lipid catabolic process | 21 | 265 | 1.58 | 6.13E-26 |
| GO:0044281 | small molecule metabolic process | 32 | 1779 | 0.93 | 1.47E-23 |
| GO:0055088 | lipid homeostasis | 15 | 111 | 1.81 | 4.01E-21 |
| GO:0010876 | lipid localization | 18 | 293 | 1.47 | 4.32E-20 |
| GO:0006641 | triglyceride metabolic process | 13 | 69 | 1.95 | 7.92E-20 |
