# Supplementary material for: ACSL4 Expression Is Associated With CD8+ T Cell Infiltration and Immune Response in Bladder Cancer
Source: Front Oncol. 2021 Nov 19;11:754845. doi: 10.3389/fonc.2021.754845 (PMC8640077; doi:10.3389/fonc.2021.754845)
Supplement: Supplementary file 3 [file Table_2.docx]

Table 2. Clinicopathological characteristics of BLCA patients with high/low ACSL4 expression level in TCGA cohort and FUSCC cohort.

| Characteristics | TCGA cohort | |  |  | FUSCC cohort | |  |
| --- | --- | --- | --- | --- | --- | --- | --- |
|  | High ACSL4  (N=204) | Low ACSL4  (N=204) |  |  | High ACSL4  (N=15) | Low ACSL4  (N=15) |  |
| N (%) |  |  |  |  |  |  |  |
| Age |  |  |  |  |  |  |  |
| ＜70 years | 99(48.5) | 180(88.2) |  |  | 7(46.7) | 10(66.7) |  |
| ≥70 years | 105(51.5) | 24(11.8) |  |  | 8(53.3) | 5(33.3) |  |
| Gender |  |  |  |  |  |  |  |
| Male | 144(72.3) | 157(77.0) |  |  | 9(60.0) | 8(53.3) |  |
| Female | 60(27.7) | 47(23.0) |  |  | 6(40.0) | 7(46.7) |  |
| Tumor stage |  |  |  |  |  |  |  |
| I-II | 58(40.2) | 74(36.3) |  |  | 13(86.7) | 1(6.7) |  |
| III-IV | 144(59.8) | 130(63.7) |  |  | 2(13.3) | 14(93.3) |  |
| T stage† |  |  |  |  |  |  |  |
| T1 – T2 | 50(27.8) | 73(37.4) |  |  | 4(36.4) | 3(20.0) |  |
| T3 – T4 | 130(72.2) | 122(62.6) |  |  | 11(63.4) | 12(80.0) |  |
| N stage† |  |  |  |  |  |  |  |
| N0-1 | 102(58.3) | 136(71.0) |  |  | 8(53.3) | 6(40.0) |  |
| N2-3 | 73(41.7) | 56(18.1) |  |  | 7(46.7) | 9(60.0) |  |
| M stage† |  |  |  |  |  |  |  |
| M0 | 55(88.7) | 141(97.2) |  |  | 14(93.3) | 12(80.0) |  |
| M1 | 7(11.3) | 4(2.7) |  |  | 1(6.7) | 3(20.0) |  |
| Subtype |  |  |  |  |  |  |  |
| Papillary | 60(29.6) | 78(52.0) |  |  | 10(66.7) | 9(60.0) |  |
| Non-Papillary | 143(70.4) | 72(48.0) |  |  | 5(33.3) | 6(40.0) |  |

† [TNM](https://en.wikipedia.org/wiki/TNM_staging_system) scoring system: Tumor size, Lymph Nodes affected, Metastases.
